# Supplementary material for: Titanium Dioxide Nanoparticles Exacerbate Allergic Airway Inflammation via TXNIP Upregulation in a Mouse Model of Asthma
Source: Int J Mol Sci. 2021 Sep 14;22(18):9924. doi: 10.3390/ijms22189924 (PMC8471251; doi:10.3390/ijms22189924)
Supplement: Supplementary file 1 [file ijms-22-09924-s001.zip › ijms-1354893-supplementary.pdf]

Titanium dioxide nanoparticles exacerbate allergic airway inflammation via TXNIP upregulation in a mouse model of asthma

Je-Oh Lim[1], Se-Jin Lee[1], Woong-Il Kim[1], So-Won Pak[1], Changjong Moon[1], In-Sik Shin[1], Jeong-Doo Heo[2*], Jong-Choon Kim[1*]

[1] College of Veterinary Medicine and BK21 FOUR Program, Chonnam National University, 77 Yongbong-ro, Buk-gu, Gwangju 61186, Republic of Korea

[2] Bioenvironmental Science & Technology Division, Korea Institute of Toxicology, Jinju 52834, Republic of Korea

[*]Correspondence: jdher@kitox.re.kr; toxkim@jnu.ac.kr

[2] Bioenvironmental Science & Technology Division, Korea Institute of Toxicology, Jinju 52834, Republic of Korea

[1] College of Veterinary Medicine and BK21 FOUR Program, Chonnam National University, 77 Yongbong-ro, Buk-gu, Gwangju 61186, Republic of Korea

Additional file 1: Figures S1−S4 and Table S1

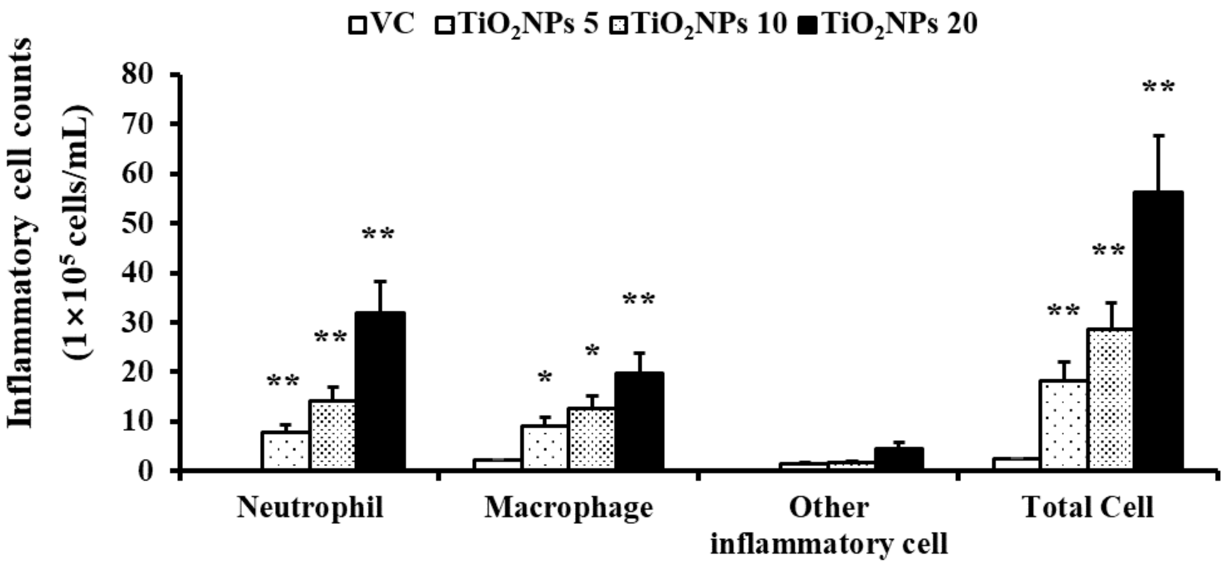

Figure S1 Effects of TiO2NPs exposure on inflammatory cell counts in BALF. Mice were exposed three times at twe-day intervals to a concentration of 5, 10, or 20 mg/kg TiO2NPs, and BALF samples were collected at 48 h after the last exposure. Exposure to TiO2NPs significantly increased inflammatory cell counts in the BALF compared with those in the VC group. VC, PBS intranasal instillation; TiO2NPs 5, 10, and 20, TiO2NPs intranasal instillation (5, 10, and 20 mg/kg, respectively). Data are represented as means ± SD, n = 6. * $p < 0.05$, ** $p < 0.01$, significantly different from the VC group.

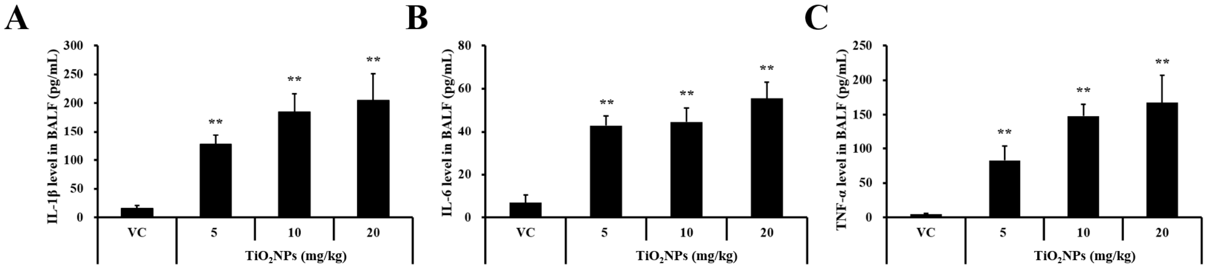

Figure S2 Effects of TiO_2NPs exposure on cytokine levels in BALF. **a** IL-1β level in BALF. **b** IL-6 level in BALF. **c** TNF-α level in BALF. The levels of the cytokines were measured in BALF using commercial enzyme-linked immunosorbent assay kits (BD Biosciences, San Jose, CA, USA) according to the manufacturer's protocol. The levels of IL-1β, IL-6, and TNF-α were significantly increased in TiO_2NPs-treated groups compared with those in the VC group. VC, PBS intranasal instillation; TiO_2NPs 5, 10, and 20, TiO_2NPs intranasal instillation (5, 10, and 20 mg/kg, respectively). Data are represented as means ± SD, n = 6. ** $p < 0.01$, significantly different from the VC group.

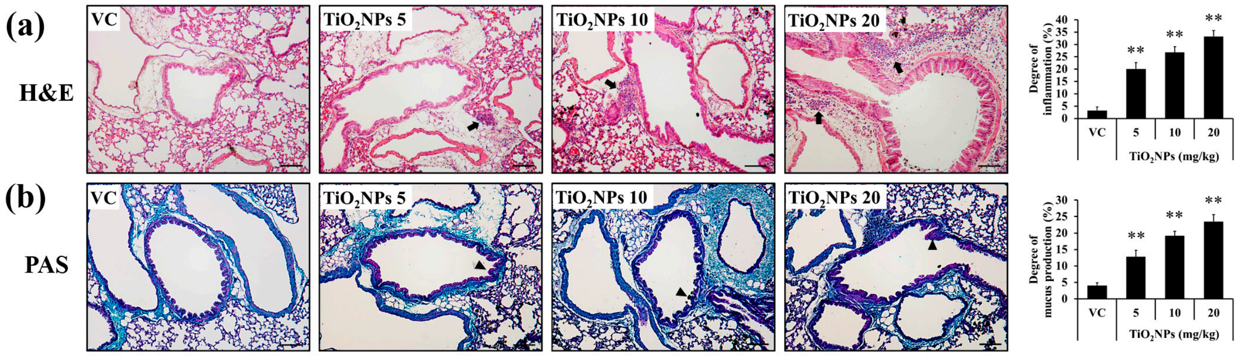

Figure S3 Effects of TiO_2NPs exposure on inflammatory cell infiltration and mucus production in the lungs. **a** Representative hematoxylin and eosin-stained sections of lung and histological scoring of inflammatory cell infiltration (× 200). **b** Representative periodic acid Schiff-stained sections of bronchi and histological scoring of mucus production (× 200). VC, PBS intranasal instillation; TiO_2NPs 5, 10, and 20, TiO_2NPs intranasal instillation (5, 10, and 20 mg/kg, respectively). Black arrows indicate inflammatory cell infiltration. Black arrowheads indicate mucus within lung epithelial goblet cells. Data are represented as means ± SD, n = 6. $^{**}p < 0.01$, significantly different from the VC group. Bar = 50 μm.

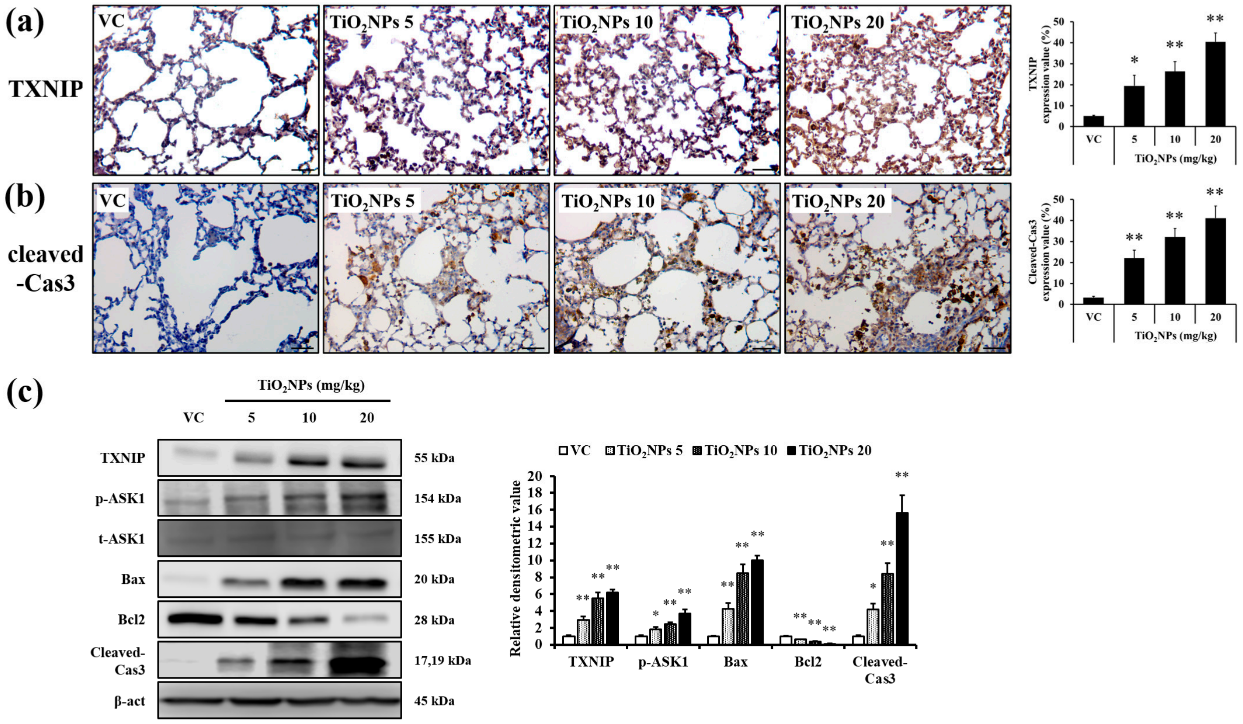

Figure S4 Effects of TiO2NPs exposure on the expression of TXNIP, p-ASK1, t-ASK1, Bax, Bcl2, and cleaved-Cas3 in the lungs. **a** Expression of TXNIP (× 400, alveolar). **b** Expression of cleaved-Cas3 (× 400, alveolar). **c** Protein expression was determined using western blotting. **d** Relative densitometric values of protein expression. VC, PBS intranasal instillation; TiO2NPs 5, 10, and 20, TiO2NPs intranasal instillation (5, 10, and 20 mg/kg, respectively). Data are represented as means ± SD, n = 6. * p < 0.05, ** *p* < 0.01, significantly different from the VC group. Bar = 50 μm.

Table S1. Primer sequences for qRT-PCR

Target genes		Sequence (5’ → 3’)	T_m°C	T_A°C
TNF-α	Forward	CAA AGT AGA CCT GCC CAG AC	59.3	55
	Reverse	GAC CTC TCT CTA ATC AGC CC	59.3	
IL-6	Forward	ATG CAA TAA CCA CCC CTG AC	57.3	55
	Reverse	ATC TGA GGT GCC CAT GCT AC	59.3	
IL-1β	Forward	AGC CAG GAC AGT CAG CTC TC	61.4	55
	Reverse	ACT TCT TGC CCC CTT TGA AT	55.2	
GAPDH	Forward	CAA AAG GGT CAT CAT CTC TG	55.2	55
	Reverse	CCT GCT TCA CCA CCT TCT TG	59.3	

TNF-α, tumor necrosis factor-alpha; IL-6, interleukin-6; IL-1β, interleukin-1β; GAPDH, glyceraldehydes-3-phosphate dehydrogenase; T_m, melting temperature of primer; and T_A, annealing temperature in PCR.
